# Adoption and income effects of new agricultural technology on family farms in China

**DOI:** 10.1371/journal.pone.0267101

**Published:** 2022-04-26

**Authors:** Fang Wu

**Affiliations:** Economics and Management School, Wuhan University, Wuhan, China; University of Palermo, ITALY

## Abstract

Family farms are the main force that promotes the direct application of new agricultural technology to production. So what are the factors that affect the adoption of new agricultural technology by family farms, and can the adoption of new technology increase the operating income of family farms? Using cross-sectional data from 847 family farms, this study examines the determinants and impacts of multiple new agricultural technologies adoption on family farms’ income in China. To account for selection bias from both observable and unobservable factors, an endogenous switching regression model is employed to evaluate the effects of new agricultural technology on family farms’ income. The empirical results show that the adoption of new agricultural technology is affected by the endowment of farmers and the characteristics of family farms. After controlling for the selection bias, the adoption of new agricultural technologies has a positive and significant impact on family farms’ income. And the impact on the non-adopter family farms is much larger than adopter family farms. Heterogeneity analysis indicates that family farms with a larger area of arable land earn more from the adoption of new technologies than small farms. In all types of technology investigated, the new methods of pest control and the new chemical fertilizer technology have a relatively large impact on family farms’ income, while the new mechanical technology has the least impact on family farms’ income. The adoption of new technologies by family farms is more important for promoting the progress of agricultural science and technology. Therefore, it is necessary to take effective measures to overcome the obstacles to the adoption of new agricultural technologies and pay more attention to the use of new agricultural technologies to improve agricultural production efficiency.

## Introduction

The Chinese government has been committed to cultivating large-scale agricultural operators, ensuring the safety of grain and agricultural industries and promoting the development of modern agriculture. As the most effective form of the new type of agricultural business, family farms(*jiating nongchang*) have pointed out the direction for the construction of Chinese modern agricultural business system [1]. Family farms are large farms worked and managed by families which have expanded significantly through state-brokered leasing of land. The government encourages the circulation of large tracts of farmland to families who devote themselves to commercial farming [2]. In recent years, family farms have developed rapidly due to the intensive promulgation of policies for fostering new agricultural business entities. Up to the end of 2018, the number of family farms is over 600,000, which is 4 times the number compared to 2013.

At the same time, many practical problems in production continue to emerge and urgently need to be resolved. The application of agricultural science and technology is a practical problem that needs to be studied and solved urgently. Science and technology is an important engine of agricultural revitalization, and family farms are the main body of a new type of agricultural production, which is at the core of the revitalization of rural industries [3]. As a consequence, it is necessary to study which factors affect the adoption behavior of agricultural technology in family farms. Farmers’ decisions on technology adoption, including their assessment of the income effect after adoption. However, most of the studies do not further examine the outcome variables of adoption behavior in the current studies on technology adoption, that is, the income effect that farmers consider when adopting. However, for farmers’ adoption decisions, technology adoption incentives and income effects are two inseparable aspects [4].

To sum up, as a producer, the family farm represents the main direction of China’s current and future advanced agricultural productivity, and is the main force to promote the direct application of new agricultural technologies to production. Family farms have both the level of demand for new technologies and the ability to adopt new technologies. It is much larger than small households. Therefore, family farms are more willing to adopt new agricultural technologies and obtain greater benefits from the adoption of new technologies [5]. However, whether the theoretical assumptions are consistent with the facts, that is, whether the adoption of new technologies can benefit family farms by bringing higher incomes, empirical tests are needed. To this end, this article uses the survey data of 847 family farms in the planting industry in my country, and uses the endogenous transformation model to explore the influencing factors of the adoption of a variety of new agricultural technologies and their impact on family farm income, and conduct robustness testing and heterogeneity analysis. Finally, it proposes countermeasures and suggestions to promote family farms to adopt new agricultural technologies.

## Literature review

With the rise and development of family farms, many literatures have conducted empirical studies on the factors affecting the adoption of new technologies in family farms. For example, relying on a field survey data from 676 family farms in Huang-huai-hai Plain, Gao et al (2017) used bivariate probit and regression linear models to explore the influence factors of the adoption of green control techniques (GCTs) by family farms. The study found that the perceived ease of use and usefulness of technology, the number of family labors, the degree of promotion in agricultural technology sector, and the education and risk preference of farmers have a significant positive impact on the willingness to adopt [6]. Ni and Liu (2019) studied the influencing factors of family farms’ application behavior of internet agricultural technology by using a survey data of 270 family farms in 9 cities in Jiangsu Province. The results show that the expected benefits, the scale of the network and online learning have a significant positive effect on the application of Internet agricultural technology by farmers [7]. Based on 2017 survey data of family farms in Heilongjiang, Jiangsu and Sichuan provinces, Xia et al (2019) found that family farmers with longer education and training are more likely to apply green production technology. However, it is not that the more time of training, the better; young, risk-preferred farmers have a higher probability of applying green production technology. Women and farmers who are accustomed to using mobile networks have a higher probability of applying green production technology [8]. Besides, Cao et al. (2020)used the ordered logistic model to explore the effects of human capital and social capital on soil testing and fertilization behavior The result shows that, overall, human capital and social capital have positive impact on the technology application of family farms [9].To sum up, although different scholars choose different explanatory variables, the adoption behavior of family farm’s agricultural technology is generally affected by farmers’ individual characteristics, family farm’s resource endowment, external production environment and other factors.

Scholars at home and abroad have also carried out a lot of research on the farmer’s income effect of agricultural technology. For example, Ma and Abdulai (2019) took 481 apple growers in China as an example. The results of the study show that the adoption of IPM technology can significantly increase apple production, net income and agricultural income [10]. Relying on 822 micro survey data of rice growers in Hubei, Jiangxi and Zhejiang provinces in the Yangtze Basin, Huang and Luo (2020) used the endogenous switching regression (ESR) to explore the cost-saving and income-increasing effect of green control techniques. The results show that: green control techniques have achieved the goal of cost-saving and income-increasing effect for rice growers, but the cost-income improvement is not large [11]. Using an endogenous switching regression model and a multinomial treatment effects mode, Gao et al (2019) evaluated the farmer’s income effect of agricultural technology, the result show that the adoption of green control techniques has a significant impact on the increase of farmer’s agricultural income [12]. Based on the micro survey data of farmers in the main apple producing areas of Shaanxi, Gansu and Henan, Hou et al (2019) employed the ESR model to examine the farmer’s agricultural income effect of technology. The results show that the soil testing formula fertilization technology helped farmers increased their average annual agricultural income by 8% [13]. Moreover, Issahaku and Abdulai (2020) used farm data from three agro-ecological regions in Ghana to examine the impact of crop selection and soil and water conservation on farm performance. The empirical result shows that farmers’ choice of crop selection and soil and water conservation is beneficial to improve crop income [14]. Tufa et al (2019) assessed the productivity and income impacts of the adoption of ISVAPs (improved soybean varieties and agronomic practices) using plot data collected from 1237 soybean farmers in Malawi. The result of the ESR model shows that the adoption of ISVAPs is related to an average 61 per cent increase in production and 53 per cent increase in income among adopters [15]. Seen from the above articles, most of the studies believe that agricultural technology adoption is beneficial to increase the household income of farmers.

The contribution of this paper to the empirical literature is threefold. Firstly, the research object of the existing literature is mainly limited to small farmers, rarely related to family farms. Small farmers have few plots, small plots, insensitive to the choice and use of technology. They are even unwilling to choose the latest technology. Different from small farmers, family farms specialize in agricultural production, with larger operation scale, stronger economic strength, and stronger willingness and ability to adopt new agricultural technologies. In the past decade, policies have been implemented to encourage the development of family farms in China. Up to the end of 2018, the number of registered family farms is 4 times the number compared to 2013.However, there are not many studies on the benefits of family farms from the adoption of new technologies, which may be due to the lack of available data.

In addition, the existing literature basically only analyzes the adoption behavior and income effects of one or two specific agricultural technologies in agricultural production, such as IPM technology [10], green control technology [11,12], green production technology [13], soil and water conservation technology [14], ISVAPs [15], water-saving irrigation technology [16], etc. Li and Kong (2010) believe that, from the perspective of the relationship between technologies, all kinds of technologies are not independent of each other, but influence and interact with each other. Only by adopting a series of related technologies at the same time can we give full play to the potential of various technologies and obtain the benefits of technological changes [17]. Different from existing research, this article discusses how a variety of new agricultural technologies, such as new varieties, new machinery, new fertilizers, new pesticides, new methods of pest control, new production and new management methods affect the income of family farms.

Secondly, whether a farm adopts new technology is affected by its own endowment, family characteristics and even the external environment, so it is not generated randomly, but a kind of "self-selection" behavior, and the problem of sample self-selection may lead to deviations in the results. Propensity Score Matching(PSM) method was used to eliminate the selectivity bias in the existing literature [18–20]. However, the PSM method solves the problem of selection bias according to observable factors. When there are unobservable factors (such as the innate ability of farmers) that affect the decision-making of new technology adoption on family farms at the same time, the PSM method may still lead to estimation bias [10]. Hence, this paper will use the endogenous switching regression (ESR) model proposed by Lokshin and sajaia (2004) [21] to make up for the defects of existing research methods. The ESR model considers the sample selection bias caused by observable and unobservable variables, which makes up for the deficiency of PSM method. In addition, the ESR model fits the income determination equations of new technology adopters and non-adopters respectively, and combines with counterfactual inference analysis, which can break through the unreasonable hypothesis that the treatment effect of two groups of family farms is homogeneous in the treatment effect model (TEM).

Thirdly, the existing literature studies on the income-increasing effect of the use of agricultural technology are either based on the macro data at the national or provincial level, or on the survey data of ordinary peasant households. However, this paper uses the micro-survey data of family farms to investigate the income-increasing effects of the new agricultural operators of family farms. It jointly adopts a variety of new agricultural technologies. The data obtained directly from the interview of family farmers are more reliable, and the sample size is relatively large. As a consequence, the conclusion should be more reliable.

The rest of this paper is organized as follows: Section 2 introduces the research method, data source and main explained and explanatory variables; Section 3 presents and discusses the empirical results; Section 3 provides the results of the robustness test and the expansive study; and the last section draws conclusions and implications for policy.

## Research method

### Endogenous switching regression model(ESR)

Whether a family farm adopts new agricultural technology depends on the difference between the utility of adopting new technology (*U*_1*i*_) and that of not adopting new technology (*U*_0*i*_)according to the random utility decision-making model of Becerril and Abdulai (2010) [22]. If $A_{i}^{*}$ = *U*_1*i*_−*U*_0*i*_>0, the family farm chooses to adopt new agricultural technology. Although the utility difference $A_{i}^{*}$ is unobservable, it can be expressed as a function of observable variables. The decision equation for the new technology of family farm is defined as:

$$A_{i}^{*}=Z_{i}\alpha +\mu_{i},\;\mathrm{if}\;A_{i}^{*}>0,\mathrm{then}A_{i}=1,\;\mathrm{otherwise}\;A_{i}=0$$ (1)

In the formula (1), $A_{i}^{*}$ indicates the unobservable latent variable of the family farm’s decision to adopt the new agricultural technology; $A_{i}$ = 1 indicates that the farmer i adopts the new agricultural technology; *A*_*i*_ = 0 indicates that the farmer i does not adopt the new agricultural technology; *Z*_*i*_ is the exogenous explanatory variable vector, that is, the variable vector that affects the family farm’s choice to adopt the new agricultural technology; *μ*_*i*_ is the random disturbance term.

The family farm management performance model can be defined as follow in order to measure the impact of new technology adoption on farm management performance:

$$Y_{i}=X_{i}\beta_{i}+\delta A_{i}+\varepsilon_{i}$$ (2)

In the formula (2), *Y*_*i*_ is the indicator of family farm management performance, such as agricultural total income and per capita income; *X*_*i*_ is the vector of individual, family and farm characteristics (such as age, education, family size, farm size, etc.) that may affect farm performance indicators; *A*_*i*_ is whether the farmer i adopts new technology variable; *ε*_*i*_ is a random disturbance term. In view of the fact that family farmers choose whether to use new agricultural technology or not according to their own conditions, the decision-making (*A*_*i*_) of new technology adoption may be affected by some unobservable factors, which are related to the result variable (*Y*_*i*_). It leads to the correlation between *A*_*i*_ and *ε*_*i*_ agriculture in formula (1). As a consequence, the direct estimation Eq (2) may lead to estimation errors due to the problem of sample self-selection.

As mentioned before, the ESR model has certain advantages in solving the problem of endogenity and comprehensively considering observable and unobservable factors, and can effectively avoid the "invisible bias" caused by unobservable factors. Therefore, we use the ESR model to examine the impact of new agricultural technology adoption on family farm operating income.

The ESR model is divided into two stages. The first stage is to use the Probit or Logit model to estimate whether the family farm adopts the new agricultural technology selection equation, as shown in Eq (1); the second stage is to establish the family farm’s operating income determination equation and estimate changes in operating income caused by the adoption of new agricultural technologies by family farms.

The management performance models of family farms with and without adopting new technology are respectively shown as follows:

$$Y_{ia}=X_{ia}\beta_{a}+\sigma_{\mu a}\lambda_{ia}+\varepsilon_{ia},\;if\;A_{i}=1$$ (2A)

$$Y_{in}=X_{in}\beta_{n}+\sigma_{\mu n}\lambda_{in}+\varepsilon_{in},\;if\;A_{i}=0$$ (2B)

In formulas (2A) and (2B), *Y*_*ia*_ and *Y*_*in*_ respectively refer to the level of management performance of family farms with and without adopting new technology. In addition, *X*_*ia*_ and *X*_*in*_ indicate the factors that affect the operating performance of the two types of family farms, as shown in Table 2; *ε*_*ia*_ and *ε*_*in*_ both represent random disturbance items. The correlation coefficients of *ε*_*ia*_, *ε*_*in*_ and *μ*_*i*_ were 1 and 0 respectively. This paper introduces the inverse Mills ratio *λ*_*ia*_, *λ*_*in*_ and their covariance $\sigma_{\mu a}=\mathrm{cov}(\mu_{i},\varepsilon_{ia})$ and $\sigma_{\mu n}=\mathrm{cov}(\mu_{i},\varepsilon_{in})$ in order to solve the problem of sample selectivity bias caused by unobservable factors. In addition, it uses the complete information maximum likelihood method to estimate the formulas (1), (2A) and (2B) simultaneously. After the estimated parameters are obtained, the net impact of the new technology adoption decision on the family farm management performance is evaluated under the counterfactual framework, that is, the average treatment effect of the new agricultural technology on the family farm agricultural operating income.

### Treatment effect estimation based on ESR model

By comparing the expected value of agricultural operating income of family farms that adopt new agricultural technologies and family farms that do not adopt new agricultural technologies under real scenarios and counterfactual scenarios, we can estimate the average processing effect of family farms adopting new agricultural technology decisions.

Expected operating income of family farms using new technologies:

$$E[Y_{ia}|A_{i}=1]=X_{ia}\beta_{a}+\sigma_{\mu a}\lambda_{ia}$$ (3)

Expected operating income of family farms without new technology:

$$E[Y_{in}|A_{i}=0]=X_{in}\beta_{n}+\sigma_{\mu n}\lambda_{in}$$ (4)

In the meanwhile, consider two counterfactual assumptions, that is, the expected operating income of a family farm using new technology without adoption:

$$E[Y_{in}|A_{i}=1]=X_{ia}\beta_{n}+\sigma_{\mu n}\lambda_{ia}$$ (5)

The expected operating income of a family farm without the use of new technology:

$$E[Y_{ia}|A_{i}=0]=X_{in}\beta_{a}+\sigma_{\mu a}\lambda_{in}$$ (6)

Through formulas (3) and (5), it is obtained that the average treatment effect of family farm operating income of new technology is as follows:

$$ATT=E[Y_{ia}|A_{i}=1]-E[Y_{in}|A_{i}=1]=X_{ia}(\beta_{a}-\beta_{n})+(\sigma_{\mu a}-\sigma_{\mu n})\lambda_{ia}$$ (7)

Similarly, the average treatment effect of operating income from family farms without new technologies is:

$$ATU=E[Y_{ia}|A_{i}=0]-E[Y_{in}|A_{i}=0]=X_{in}(\beta_{a}-\beta_{n})+(\sigma_{\mu a}-\sigma_{\mu n})\lambda_{in}$$ (8)

## Data source

The data in this paper come from the three-round Family Farm Survey during 2017~2019 conducted by the Family Farm Research Group of the Economic Development Research Center of Wuhan University. The first round was carried out in 2017 in Wuhan City, Hubei Province and Langxi County, Anhui Province. The second round, fielded in 2018, took place in 20 provinces (districts, municipalities) across China. The third round was carried out in 2019 in Wuxue City and Macheng City, Hubei Province. The subjects of the three-round survey are all local family farmers engaged in agricultural production. The rosters of family farmers were provided in advance by the local agricultural management departments such as the Bureau of Agriculture. Then we conducted a random sampling survey based on the roster. The three-round survey employs the same questionnaire through face-to-face interview. A total sample of 1,373 family farms is obtained. After excluding the missing values and outlier, we obtain the data with 1,324 observations, 847 of which are planting-based. The questionnaire includes questions about the basic information of the farmer’s family, land circulation and utilization, fixed assets and investment, labor and wage, income and expenditure of production and operation, new technology demand and application, natural and market risks, cooperative membership and leading enterprises, finance and insurance, and other related issues. We focus on 847 planting family farms in this paper.

In the survey sample of family farms (Table 1), 69.54% of the family farm operators are aged 36–55; the majority of family farm operators have an education level of junior high school, accounting for 44.98%; the number of family members is mostly 3–4 people, accounting for 58.56%; family farms with an operating scale of more than 50 mu(3.335 hectare), accounted for 83.59%; family farms with operating years of 20 to 30 years accounted for the largest proportion, accounting for 41.32%; family farms with an operating income of less than 500,000 yuan($78,950),accounted for the vast majority, accounting for 61.63%.

**Table 1:** Basic characteristics of the sample family farms.

Variable	Category	Number of farms	Percentage (%)	Variable	Category	Number of farms	Percentage (%)
Age	≦35	45	5.31	Education	Elementary school and below	131	15.47
	[36,45]	180	21.25		junior high school	381	44.98
	[46,55]	409	48.29		high school	234	27.63
	>55	213	25.15		College and above	101	11.92
Family members	≦2	125	14.76	Years of farming	≦10	273	32.23
	[3,4]	496	58.56		[10,20]	251	29.63
	[5,6]	207	24.44		[20,30]	350	41.32
	>6	19	2.24		>30	27	3.19
Farm size(mu)	≦50	139	16.41	Farm income(yuan)	≦50	522	61.63
	(50,150]	241	28.45		(50,100]	142	16.77
	(150,250]	164	19.36		(100,150]	55	6.49
	(250,350]	103	12.16		(150,200]	40	4.72
	>350	200	23.61		>200	88	10.39

Note: 1 yuan = 0.16 USD in January, 2022; 1 mu = 1/15 hectare

### Variables and descriptive statistics

#### Explained variables

The explained variables are the total operating income and the per capita operating income of family farms. The total operating income of family farms is based on the question in the questionnaire: “How many tens of thousands of yuan was the total operating income of your family farm last year?” In addition, there are great differences in the number of workers involved in agricultural production on family farms(Some farms have only one person engaged in agricultural production, and some even as many as nine people engaged in agricultural production.). As a consequence, it is not enough to take the total income of the family farm as the explanatory variable. We also take the per capita income of the family farm labor force as the explanatory variable. Per capita income is a better reflection of the labor productivity of a family farm. The per capita operating income of the family farm is obtained by dividing the total operating income of the family farm by the number of own labor. In order to reduce the impact of outliers and heteroscedasticity, this paper has carried out a logarithmic treatment on family farms’ operating income.

#### Core explanatory variable

The core explanatory variable in the study is a dummy variable that equals one if the family farm adopts any new technology, and equals zero if not. This variable is based on the question in the questionnaire: "Have you used the following new technologies in the past year?" (you can choose more) ", the alternative options are" 1. New varieties(new agricultural varieties such as rice, wheat, corn, rape and soybean); 2. New machinery (agricultural machines such as rice transplanter, planter, harvester, dryer, agricultural tractor); 3. New chemical fertilizers; 4. New pesticides; 5. New pest control methods; 6. New production methods (e.g. new crop cultivation methods, new fertilization formulations, new pesticide spraying methods, new irrigation methods, etc.);7. New management methods (e.g. new field management, new operating methods, new cost control methods, etc.); 8. Did not use any new technology. If the farmer chooses 8, it is considered that the farm does not adopt new technology, with a value of 0. Otherwise, it is considered that new technology is adopted, with a value of 1.

Table 2 shows the adoption of new technology types in family farms.

**Table 2:** Statistics of new agricultural technologies adopted by family farms.

Technology category	New varieties	New machinery	New fertilizers	New pesticides	New pest control methods	New production methods	New management methods	None
Number of samples (units)	398	173	254	332	236	234	157	190
Percentage (%)	46.99	20.43	29.99	39.20	27.86	27.62	18.54	22.42

As can be seen from Table 3, the proportion of sample family farms using two new technologies is the largest, accounting for 22.67%, followed by one and three, accounting for 19.01% and 15.70%, respectively. Family farms using five or more new technologies account for relatively few.

**Table 3:** Statistics on the number of new agricultural technologies adopted by family farms.

Number of technologies adopted	1	2	3	4	5	6	7
Number of households adopted	161	192	133	85	37	28	21
Percentage (%)	19.01	22.67	15.70	10.04	4.37	3.31	2.48

#### Control variables

Similar to earlier studies [10–15], we employ three types of control variables, which are personal characteristics of head of family farm, farm operation characteristics and external environment characteristics. The personal characteristics of head of family farm include age, education, years of farming, past career and technology training experience. The characteristics of farm operation include family labors, farm size, contract farming, internet use and whether it is a model family farm. The external environment characteristics include the availability of loans, cooperative membership, and government extensions. In addition, the effects of different years and regions on the adoption of new technologies and operating income of family farms are taken into account in the estimation of the model.

#### Instrumental variable

Similar to earlier studies [10,15,19,23], "Whether the farmer can obtain information about new agricultural technologies from television, radio, newspapers, magazines and other channels" was selected as an instrumental variable. For the validity check of this instrument, we have run simple probit model for selection equation and OLS regression for outcome equations separately and we have checked that this variable is significant when included in the adoption of new technology selection equation but not significant when included in the outcome equations [10]. Then, following Di Falco et al. (2011) and Kumar et al. (2018), we conduct further falsification test [23,24]. The results show that the selected IV has a significant positive impact on the decision of family farms to adopt new technology but has no impact on the operating income of family farms that do not adopt new technology (see Appendix Table A.I). The above results together show that this IV is indeed an effective instrumental variable.

The definition and descriptive statistics of variables used in empirical analysis are shown in Table 4.

**Table 4:** Variable definition and descriptive statistics.

Variables	Variable definition	Mean	Std. Dev.
Dependent variables			
Total income	Total operating income of family farm in one year (ten thousand yuan)	99.68	219.22
Per capita income	Total operating income divided by number of family labors in agriculture (ten thousand yuan per capita)	53.44	155.98
Independent variable			
Adoption of new technology	1 if family farm has adopted any new technology, 0 otherwise	0.78	0.42
Control variables			
Age	Age of head of family farm	49.80	8.24
Education	Illiteracy = 1, primary school = 2, junior high school = 3, high school or equal = 4, junior college or equal = 5, college or above = 6	3.37	1.00
Years of farming	Years that family farm has been engaged in agricultural production	20.81	12.20
Past career	1 if the head of family farm was a farmer before he set up the farm, 0 otherwise	0.81	0.39
Training experience	1 if the head of family farm has attended any technical training, 0 otherwise	0.68	0.47
Family labors	Number of family members engaged in agriculture	2.26	0.97
Farm size	Total farming area (mu)	312.08	462.98
Loans	1 if family farm has agricultural loans, 0 otherwise	0.59	0.49
Internet use	1 if the head of family farm often uses Internet for operation, 2 if sometimes, 3 if never	1.76	0.77
Contract farming	1 if the family farm signs contracts with leading agricultural enterprises, 0 otherwise	0.19	0.40
Cooperative membership	1 if the family farm participates in cooperative, 0 otherwise	0.38	0.49
Model family farm	1 if the family farm is a model farm, 0 otherwise	0.40	0.49
Government extensions	1 if the family farm has extension service from government, 0 otherwise	0.84	0.25
Instrumental variable			
Access to information	1 if the family farm gets information about new agricultural technologies from TV, radio, newspapers, magazines and others, 0 otherwise	0.23	0.42

Note: 1 yuan = 0.16 USD in January, 2022; 1 mu = 1/15 hectare

*p<0.1

**p<0.05

***p<0.01;Farm size and family farms’ income take the natural logarithm in the empirical study; Limited to space, the results of statistical analysis of region and year dummy variables are not reported.

The descriptive statistics provided in Table 5 show that there are systematic differences between adopters and non-adopters of new technologies in terms of observable characteristics at the individual, family and farm levels. In particular, at a given level of significance, the operating income of family farms using new agricultural technologies is also higher. For example, the average annual operating income of family farms using new technologies is 1.06 million yuan ($158,679). The average annual operating income of family farms without new technologies is 0.79 million yuan($117,846), with a difference of 0.27 million yuan($40,844). However, given that the adoption of new technology is the result of "self-choice" of family farms, the differences in agricultural operating income between adopters and non-adopters of new technologies listed in Table 5 are not sufficient to explain the causal relationship between the adoption of new technologies and family farms’ income. As a consequence, this paper uses the ESR model to assess the impact of new technology adoption behavior on family farms’ income. It can correct selection biases and endogenesis from observed and unobserved heterogeneity.

**Table 5:** Mean differences in characteristics between new technology adopters and non-adopters.

Variables	Adopters(n = 657)	Non-adopters(n = 190)	Diff
Age	49.265	51.653	-2.388***
Education	3.381	3.316	0.065
Farming experience	20.329	22.458	-2.129**
Past career	0.802	0.826	-0.024
Training experience	0.706	0.600	0.1068***
Family labors	2.268	2.216	0.052
Farm size	323.553	272.417	52.137
Internet use	1.680	2.011	-0.3308***
Loans	0.613	0.495	0.1198***
Cooperative membership	0.412	0.268	0.1448***
Contract farming	0.224	0.089	0.1348***
Government extensions	0.951	0.879	0.0728***
Model family farm	0.425	0.311	0.1148***
Access to information	0.307	0.058	0.2498***
Total income	105.786(3.803)	78.564(3.418)	27.222(0.3848***)
Per capita income	53.807(3.112)	52.180(2.779)	1.627(0.3348***)

Note: 1 yuan = 0.16 USD in January, 2022; 1 mu = 1/15 hectare

*p<0.1

**p<0.05

***p<0.01; the natural logarithm of family farms’ income (total income and per capital income)are taken in parentheses.

## Empirical results and analysis

### The selection and outcome models

As we can see from Table 6, it shows the results of the ESR model estimation of the impact of the adoption of new technologies on the operating income indicators of family farms. The lower half of Table 6 shows that the estimated values of the correlation coefficient rho_1/rho_2 in the model are significantly non-zero, indicating that there is a sample selection bias caused by unobservable factors. In particular, the negative correlation coefficient of rho_1/rho_2 indicates the negative selection deviation. This shows that family farms with lower total operating income and per capita operating income are more likely to adopt new agricultural technologies. If this negative selection bias cannot be explained, the impact of the adoption of new technologies on family farms’ income will be underestimated. The LR test values of equation independence reject the original hypothesis that the selection equation and the result equation are independent of each other at the statistical level of 1%. The goodness-of-fit test of the model is significant at 1% level. As a consequence, the ESR model used in this paper is appropriate.

**Table 6:** Impact of the adoption of new technologies on the operating income of family farms: Estimated by the ESR model.

Variables	Adoption of New technology	Model 1 (logarithm of total income)		Adoption of New technology	Model 1 (logarithm of per capita income)	
		Adopters	Non-adopters		Adopters	Non-adopters
Age	-0.016**(0.008)	-0.004(0.006)	-0.015(0.012)	-0.017** (0.008)	-0.005(0.006)	-0.014(0.011)
Education	-0.081(0.056)	0.131***(0.047)	-0.105(0.090)	-0.079(0.057)	0.129***(0.044)	-0.097(0.085)
Farming experience	0.000 (0.005)	-0.000(0.004)	0.011(0.007)	0.002 (0.005)	-0.001(0.004)	0.010(0.007)
Past career	-0.123 (0.141)	-0.096(0.111)	-0.030(0.216)	-0.117 (0.145)	-0.097(0.103)	-0.045(0.204)
Training experience	0.350***(0.117)	0.005(0.101)	-0.012(0.215)	0.343***(0.119)	0.003(0.094)	0.010(0.199)
Family labors	-0.003(0.055)	0.155***(0.044)	-0.039(0.080)	-0.012 (0.055)	-0.235***(0.041)	-0.394***(0.075)
Farm size	-0.056(0.048)	0.560***(0.040)	0.176**(0.087)	-0.054(0.050)	0.558***(0.037)	0.195**(0.083)
Internet use	-0.148**(0.070)	-0.009(0.059)	-0.102(0.125)	-0.139*(0.072)	-0.021(0.056)	-0.118(0.116)
Loans	0.225**(0.105)	-0.033(0.086)	0.394**(0.192)	0.213**(0.107)	-0.025(0.080)	0.385**(0.179)
Cooperative membership	0.321***(0.114)	0.030(0.090)	0.200(0.204)	0.323***(0.116)	0.032(0.084)	0.197(0.192)
Contract farming	0.440***(0.156)	0.140(0.105)	1.073***(0.316)	0.448***(0.159)	0.136(0.098)	0.988***(0.298)
Government extensions	0.424**(0.185)	-0.178(0.186)	0.162(0.278)	0.432**(0.188)	-0.132(0.176)	0.149(0.262)
Model family farm	0.140(0.119)	0.258***(0.098)	0.688***(0.182)	0.158(0.122)	0.257***(0.091)	0.603***(0.173)
Access to information	1.034***(0.177)			1.112***(0.183)		
Constant	1.191**(0.543)	0.496(0.462)	2.597**(1.122)	1.223***(0.556)	0.699(0.430)	2.773***(1.054)
Region	Yes	Yes	Yes	Yes	Yes	Yes
Year	Yes	Yes	Yes	Yes	Yes	Yes
rho_1	-0.816***(0.059)	rho_2	-0.160(0.469)	-0.725***(0.090)	rho_2	-0.101(0.459)
Wald chi2	373.29***	Log likelihood	-1543.12	408.52***	Log likelihood	-1501.68
LR test of indep. eqns.		chi2(1) = 14.11,Prob > chi2 = 0.0002		chi2(1) = 9.32, Prob > chi2 = 0.0023		

Note

*p<0.1

**p<0.05

***p<0.01; Standard errors in parentheses.

The results of the first stage estimation of the ESR model show the determinants of the adoption of new technologies in family farms. The results in Table 6 show that the age of the household head is negative and significant at the 5% statistical level, which indicates that the older the farmer, the less likely it is to adopt new agricultural technologies. This is because the older farmers are, the more likely they are to be influenced by traditional habits and tend to be more resistant to new technologies. Family farmers who have training experience are significantly more likely to adopt new technologies. This is because training experience as a way of education, by imparting experience to farmers, let farmers understand and master the use and economic value of new technology, and promote the adoption of new technology by farmers. Family farms invest a lot in the initial stage of construction and often lack their own funds. However, the use of some new technologies requires greater investment, so there is a greater demand for funds. The empirical results show that the status of fund borrowing is significantly positive at the 5% level, indicating that the availability of borrowing has a significant positive impact on the adoption of new technologies by family farms, which is consistent with the research conclusions of Gao et al. (2017,2019) [6,12]. The coefficient of cooperative membership is positive and significant at the 1% level, which indicates that family farms participating in the cooperative are more likely to adopt new agricultural technologies. This is because farmers’ cooperatives can reduce the high-risk and high-cost dilemmas faced by members in using the elements of new technologies and promote the adoption of new technologies [10,25]. Accessing information online has a significant positive impact (at the 5% statistical level) on the adoption of new technologies by family farms. The using of Internet can alleviate the information constraints of family farmers and improve their ability to obtain information. Information flow can improve farmers’ understanding and mastery of technological information and promote farmers to adopt new technologies [26]. Signing purchase and sales contracts with companies has a significant positive impact(at the 1% statistical level) on farmers’ willingness to adopt new technologies. That is because contract agriculture provides farmers with services such as credit purchase of means of production, credit collateral, technical guidance and training, and information provision. It can help farmers reduce credit constraints and transaction costs for adopting new technologies [20]. The coefficient of government extension services is positive and significant at the 5% level, indicating that government extension services have a significant role in promoting the adoption of new technologies by family farms The promotion and publicity of new technology by government departments can effectively improve farmers’ awareness of new technology. Besides, technology subsidies provided by the government to farmers who adopt new technologies can also increase their enthusiasm for adopting new technologies [27]. Finally, the coefficient of the instrumental variable "Access to information" is positive and significant at the 1% level. This indicates that the availability of new technology information has a significant impact on family farms’ decision to adopt new agricultural technologies. Some studies have pointed out that the wider the channels for farmers to obtain technical information, the more adequate the relevant technology information will be, the greater the possibility of using relevant technologies [6,12].

The results of the second stage estimation of the ESR model show the determinants of family farm operating income. As can be seen from the result equation in Table 6, there are significant differences in coefficient estimates between the two types of family farms with and without new technology in some variables. For example, the effect of education level is significantly positive at the 1% level, which shows that with the improvement of farmers’ human capital, it is helpful to promote the increase of their operating income. The impact of family labors on farm economic benefits is significantly positive at the 1% level. Generally speaking, family farms with more self-owned labor force are more willing to invest more labor force in the process of agricultural production so as to obtain higher land income. However, too many farmers in the family will reduce the per capita operating income of the farm. From the estimated results in Table 6, it can be seen that regardless of whether new agricultural technologies are adopted, the coefficient of farm scale is positive and significant at the 1% level. This shows that relatively large-scale farms are more likely to drive farms to achieve economic benefits. Loans and contract farming have a significant positive impact (at the statistical levels of 5% and 1%, respectively) on the operating income of family farms that do not adopt new technology. The former is because the difficulty of financing and the shortage of production funds are the outstanding problems faced by family farms. However, farmers can ease the liquidity constraints in the construction of agricultural infrastructure, the purchase of machinery and equipment, and the application of advanced agricultural technology through loans, which can increase production investment and promote income growth. The latter is because when agricultural leading enterprises sign contracts with family farms for the purchase and sale of agricultural products, they usually set a series of quality standards and provide family farms with high-quality inputs and corresponding production technologies, thus stabilizing and improving product quality and output, as well as raising the production technical efficiency and income level of family farms [28]. Finally, it can be seen from Table 6 that regardless of whether the new agricultural technology is adopted or not, the coefficient of the model family farm is significantly positive at the level of 1%, indicating that obtaining the model certification has a significant and positive impact on the income(both total income and per capita income) of the family farm This is because family farms that have been certified for demonstration have strong standard demonstration significance and economic driving capabilities in terms of basic equipment, production and operation, production efficiency, and demonstration driving, which can significantly promote family farms to quickly improve economic benefits and promote families rapid increase in farm value and efficiency [18].

### Estimating treatment effects

The following further compares the expected income of the family farm (a) with the new technology and the family farm(b) without the new technology. In addition, we intend to compare the family farms with new technology if they do not adopt (c) and the family farms without new technology if they adopt (d), namely the expected income under the actual situation and the counterfactual conditions. The results are shown in Table 7. We take the average treatment effect of the impact of new technologies on the total income of family farms as an example (per capita income can be similarly explained as a result variable). The expected income of the family farm that actually adopts the new technology is 3.809. The expected income of the family farm that does not adopt the new technology is 3.573 under the counterfactual condition. The difference between the two reflects the average treatment effect of the new technology, that is, the income-increasing effect brought by the use of the new technology. The average treatment effect (ATT) is 0.236, indicating that farms with new technologies will reduce their expected income by 6.20%(The calculation formula is:(3.573–3.809)/3.809*100%). if they are not adopted. Similarly, if farms that do not actually adopt new technologies (b and d) adopt new technologies, the average treatment effect (ATU) is 1.533, indicating that expected income will increase by 44.85%(The calculation formula is:(4.951–3.418)/3.418*100%). As a result, adoption of new farm technologies has a positive effect on the family farms’ income, which is significant at the 1% statistical level. It can also be seen from Table 7 that family farms that do not actually adopt new technologies will have a more significant increase in income if they adopt new technologies (ATU > ATT). As a consequence, a basic policy implication is that family farms that do not adopt new technologies should be encouraged to adopt new technologies at present.

**Table 7:** Average treatment effect of the impact of new technology adoptation on family farms’ operating income.

Outcomes	Mean outcome		ATT	ATU	t-Value
	Adopters	Non-adopters			
Total income	(a)3.809(0.033)	(c)3.573(0.032)	0.236***(0.026)		9.25
	(d)4.951(0.061)	(b)3.418(0.050)		1.533***(0.037)	41.41
Per capita income	(a)3.116(0.031)	(c)3.005(0.032)	0.110***(0.024)		4.64
	(d)4.022(0.058)	(b)2.779(0.053)		1.243***(0.034)	36.42

Note: Standard errors in parentheses

*p<0.1

**p<0.05

***p<0.01; ATT and ATU refer to the average treatment effects of farms with new technology and farms without new technology respectively. As the outcome variables in ESR outcome equations are the logs of family farms’ total and per capita income, the results of the mean outcomes of both groups and the predictions of ATT and ATU are in log forms.Same below.

## Robustness test and heterogeneous effects

### Robustness test

In order to solve the endogenous problem between the adoption of new agricultural technology and the income from family farm operations, the article further uses the endogenous treatment effect model (ETEM) for quantitative analysis as a robustness test [10]. The model can estimate two equations at the same time: the selection Eq (1) and the outcome Eq (2). The former examines the factors influencing if the farm chooses to adopt new technologies; the latter analyzes the influence of whether to adopt new technologies and other factors on farm operating income. The ETEM estimation results reflect the marginal impact of never adopting new technologies into adopting new technologies on the income of family farms.

The results estimated by the ETEM model show that the adoption of new technology has a significant positive effect on farm agricultural operating income and per capita operating income (see Table 8) after controlling other variables. It indicates that the adoption of new technology can indeed increase family farm operating income. Except that the instrumental variable "Access to information" has a significant positive effect on the adoption of new technology on farms, training experience, internet use, loans, cooperative membership, contract farming, and government extensions have also passed the significance test in the selection equation. Age and education level have significant negative effects on the adoption of new technology on farms. The reasons have been explained earlier, so we will not repeat them. Other variables such as Past career, the number of family farmers, the number of years of farming, the size of the farm, whether it is a demonstration farm or not have no significant impact on the adoption of new technology on the farm. In the result equation, there is an obvious negative relationship between age and farm economic benefits. The number of family farmers, the size of the farm, whether to sign the contract for the purchase and sale of agricultural products, and whether it is a model farm have a significant positive impact on farm income. Similar to the regression results of the ESR model above, from the regression results of these variables, we can make practical policy recommendations for promoting the adoption of new technologies for family farms.

**Table 8:** Impact of the adoption of new technologies on the operating income of family farms: Estimated by the TEM model.

Variables	Model 1		Model 2	
	Adoption of New technology	logarithm of total income	Adoption of New technology	logarithm of Per capita income
Adoption of New technology		0.660**(0.323)		0.613**(0.294)
Age	-0.021***(0.008)	-0.010*(0.006)	-0.021***(0.008)	-0.009*(0.005)
Education	-0.098*(0.059)	0.065(0.041)	-0.098*(0.059)	0.072*(0.039)
Years of farming	0.005(0.005)	0.003(0.004)	0.005(0.005)	0.002(0.003)
Past career	-0.075(0.147)	-0.091(0.096)	-0.078(0.147)	-0.090(0.092)
Training experience	0.327***(0.123)	0.010(0.088)	0.329***(0.123)	0.010(0.083)
Family labors	-0.030(0.057)	0.113***(0.038)	-0.028(0.056)	-0.268***(0.036)
Farm size	-0.066(0.054)	0.502***(0.035)	-0.067(0.054)	0.503***(0.033)
Internet use	-0.136*(0.073)	-0.074(0.055)	-0.135*(0.073)	-0.073(0.052)
Loans	0.185*(0.109)	0.053(0.076)	0.185*(0.109)	0.049(0.072)
Cooperative membership	0.344***(0.118)	0.088(0.081)	0.345***(0.118)	0.076(0.076)
Contract farming	0.500***(0.163)	0.292***(0.097)	0.498***(0.163)	0.260***(0.092)
Government extensions	0.463**(0.191)	-0.006(0.153)	0.462**(0.191)	-0.012(0.145)
Model family farm	0.171(0.124)	0.354***(0.084)	0.169(0.124)	0.324***(0.080)
Access to information	1.142***(0.188)		1.150***(0.187)	
Constant	1.488***(0.573)	0.292(0.494)	1.492***(0.572)	0.509(0.463)
Region	Yes		Yes	
Year	Yes		Yes	
rho	-0.350**(0.180)		-0.353**(0.172)	
lambda	-0.352*(0.192)		-0.337*(0.174)	
LR test of indep. eqns. (rho = 0)	chi2(1) = 3.09, Prob > chi2 = 0.079		chi2(1) = 3.34, Prob > chi2 = 0.068	

Note

*p<0.1

**p<0.05

***p<0.01; Standard errors in parentheses.

### Heterogeneous effects

#### Different farm sizes

Family farm is a new type of management body that the central government focuses on encouraging and supporting development. Family farm will become the main management body of agriculture in our country and the main force in the demand and adoption of new agricultural technology. From the perspective of expected income, compared with traditional small farmers, the operating scale of family farms is ten times or even hundreds of times larger than that of small farmers. As a consequence, the potential income of new technologies is much higher than that of small farmers. However, this paper is more concerned about whether the larger the scale of operation, the greater the potential benefits of the adoption of new technology for family farms that have achieved moderate scale operation. We first calculate the mean value of the cultivated land area variable of family farm management in order to investigate whether there is a difference in the impact of the adoption of new agricultural technology on family farm income under the comparison of larger and smaller business scale. So, the samples are divided into two sample groups: "greater than the average" and "less than the average" for comparative analysis. Table 9 the results of ESR model estimation show that after controlling other variables, the adoption of new agricultural technology has a significant positive effect on the total income of family farm. The impact of the adoption of new agricultural technology on family farms’ income is 2.757 in the "greater than the average" sample group and only 0.227 in the "less than the average" sample group. This shows that the larger the operating area of land, the greater the income-increasing effect brought about by the adoption of new technology on family farms.

**Table 9:** Impact of new technology adoption on family farms’ income under different operating scales: Estimated by ESR model.

Category	Mean outcome		ATT	ATU	t-Value
	Adopters	Non-adopters			
Greater than the average	4.782(0.053)	2.025(0.067)	2.757***(0.068)		40.72
	5.971(0.125)	4.185(0.167)		1.786***(0.165)	10.82
Less than the average	3.409(0.027)	3.183(0.308)	0.227***(0.027)		8.28
	4.524(0.046)	3.224(0.046)		1.300***(0.039)	33.22

#### Different types of new technologies

We make a group analysis according to the type of agricultural technology in order to investigate whether there are differences in the impact of different agricultural technology adoption on family farm income. From the estimated results in Table 10, compared with other technical types, the income-increasing effect of the new methods of pest control and new chemical fertilizer technology is relatively more obvious. On the one hand, the new method of pest control can reduce the yield loss of agricultural products and bring positive benefits to family farms when the output value spills over the price of pesticides. On the other hand, the demand for high-quality, safe and hygienic edible agricultural products is increasing day by day with the rapid development of economy and the continuous improvement of people’s living standards.

**Table 10:** Effects of different new agricultural technologies on family farms’ income: Estimates by ESR model.

Category		Mean outcome		ATT	ATU
		Adopters	Non-adopters		
New varieties	Yes	3.808(0.046)	3.449(0.037)	0.359***(0.021)	
	No	5.194(0.039)	3.634(0.033)		1.560***(0.019)
New machinery	Yes	4.189(0.071)	3.922(0.057)	0.267***(0.033)	
	No	5.200(0.031)	3.594(0.028)		1.606***(0.015)
New fertilizers	Yes	3.694(0.056)	3.310(0.048)	0.384***(0.026)	
	No	5.349(0.037)	3.726(0.032)		1.623***(0.020)
New pesticides	Yes	3.666(0.049)	3.293(0.044)	0.373***(0.017)	
	No	5.492(0.037)	3.747(0.033)		1.745***(0.015)
New pest control methods	Yes	4.079(0.058)	3.667(0.052)	0.412***(0.030)	
	No	5.780(0.030)	3.578(0.030)		2.202***(0.021)
New production methods	Yes	4.094(0.055)	3.753(0.048)	0.341***(0.025)	
	No	5.623(0.034)	3.575(0.030)		2.048***(0.015)
New management methods	Yes	4.123(0.064)	3.811(0.063)	0.312***(0.026)	
	No	4.001(0.029)	3.624(0.029)		0.377***(0.013)

The adoption of new methods of pest control will weaken the impact on the quality and safety of agricultural products caused by excessive use of pesticides and then affect the price, thus reducing the negative impact of excessive use of pesticides on the income of family farms. In addition, the adoption of new methods of pest control can also save the use of agricultural labor, reduce the labor input cost of family farms, and thus increase the income of family farms. As for the adoption of new chemical fertilizer technologies, such as the use of organic fertilizers can not only provide nutrition for crops, but also increase and renew soil organic matter. In addition, it can promote microbial reproduction, and improve soil properties and biological activities, thereby increasing soil fertility and fertilizer use efficiency. It helps to increase crop yields and improve the quality of agricultural products, thereby increasing the income of family farms.

The adoption of new pesticides and new varieties of technology have a great impact on the income of family farms. The promotion and application of high-efficiency, low-toxicity, safe and high-quality pesticides in a large area has promoted a bumper agricultural harvest. New varieties can enrich the variety of agricultural products. In addition, the diversification of agricultural products makes family farms have more production options, thus increasing new income channels of agricultural production. New production methods, new management methods are significantly positive at 1% statistical level. It indicates that new production methods and new management methods can promote family farms to increase production and income. The new mechanical technology has the least impact on the income of family farms, which may be related to the low degree of mechanization and limited role of family farms in the survey area.

## Conclusion and policy implications

China agriculture is still dominated by small-scale farmers, but their demand for new technologies is insufficient. Family farms of scale operators are mainly engaged in agricultural production and are far more willing to adopt new technologies than smallholder farmers. Relying on cross-sectional data from 847 family farms, this study examines the determinants of adoption of multiple new agricultural technologies and its impact on family farms’ income in China. The empirical results show that agricultural technical training, loan availability, internet use, participation in cooperatives, contract farming, government extension services and access to new technology information are main factors that determine farmers’ decision to adopt new agricultural technology, and new agricultural technology adoption has a positive and statistically significant impact on family farms’ income. Under the counterfactual assumption, if the family farms that actually adopt the new technology do not adopt the new technology, the total income of family farms will decrease by 6.20%. If the farms that do not adopt the new technology adopt the new technology, the total agricultural operating income will increase by 44.85%. In addition, the robustness test from the ETEM model also supports the above conclusions. The heterogeneity study found that, the larger the land management area is, the greater the income increase effect brought by the use of new technology is. And the income increase effect of using new methods of pest control and new chemical fertilizer technology is relatively more obvious.

The empirical results of this paper support the view that increasing the adoption of new agricultural technologies will help to improve the economic benefits of family farms. As a consequence, it is necessary to take a variety of effective measures to support family farms to carry out the application of new agricultural technologies and to promote family farms to increase production and income: (1) When researching and developing new agricultural technologies, it is necessary to have a deep understanding of the needs of agricultural producers, so as to match the new technologies developed with their needs and improve the realization of new technologies. (2) By establishing a family farms’ agricultural new technology demand feedback and service evaluation mechanism, forming a farmer’s demand-oriented agricultural new technology service system, rationally providing the agricultural new technology required by farmers, will reduce the supply cost of agricultural new technology services and increase the adoption rate of new technology.(3)Encourage family farms to join agricultural cooperatives, and use cooperatives as a platform and mechanism for regular exchanges between farmers. Through technical guidance and experience exchange, new agricultural technologies can be more smoothly promoted among family farms adjacent to geographical space. (3) The government should expand the service functions of township agricultural technology extension organizations. In addition to teaching new technologies to farmers, the agricultural technology department should also organize farmers to observe and learn from family farms that have adopted new technologies in nearby towns, build farmers’ new technology information exchange platform, and improve the adoption rate of new technologies in a wider geographical range. (4) Carefully select and cultivate new technology promotion demonstration households. Farmers with younger age and higher education level are more willing to adopt new technologies. Agricultural technology extension agencies should select young farmers with strong technology acceptance ability as model households for new technology adoption, in the whole process of new technology application. Give vigorous support and help to improve the success rate of new technology adoption by model households, and promote new agricultural technologies through demonstration effects.

Different from existing research, this article discusses how a variety of new agricultural technologies affect the income of family farms. This is because, in reality, farmers rarely use one agricultural technology, but use a combination of agricultural technologies. We provide supportive evidence to the causal relationship between new agricultural technologies’ adoptoin and family farms’ income. We believe that our research provides an important case study and empirical result to the previous research. Our analysis is based on cross-sectional data collected from 847 family farms in China. Due to data limitations, we are unable to capture the dynamic effects of multiple new agricultural technologies adoption on farm performance. However, we believe this is a promising area to investigate in the future when the required panel data are available.

### Appendix

**Table A.I:** Test of the validity for the selection instrument.

Variables	Model 1	Model 2	Model 3	Model 4	Model 5
	Adoption of New technology	Total income	Income per capita	Total income of Non-adopters	Income per capita of Non-adopters
Instrument Variable	1.148***(0.196)	0.133(0.088)	0.135(0.086)	0.172(0.229)	0.117(0.211)
Control variables	Yes	Yes	Yes	Yes	Yes
Region	Yes	Yes	Yes	Yes	Yes
Year	Yes	Yes	Yes	Yes	Yes
Constant	1.435**(0.581)	0.873**(0.445)	1.046**(0.418)	2.869**(1.175)	2.938***(1.091)
Observations	847	847	847	190	190

Note: The values in parentheses are robust standard errors; *p<0.1

**p<0.05

***p<0.01; the control variables are the same as those used in the previous ESR model.

## Supporting information

S1 Data (XLSX)
